# Corneal higher-order aberrations as key predictive indicators of axial elongation in myopic children with orthokeratology: a single-center prospective cohort study

**DOI:** 10.1038/s41598-025-17115-w

**Published:** 2025-08-23

**Authors:** Ju Zhang, Xiaoliang Zhang, Ranyi Ding, Yanan Ji, Zhe Zhu, Qingdong Bao, Ting Wang, Weiyun Shi

**Affiliations:** 1 Shandong First Medical University and Shandong Academy of Medical Sciences, Jinan, Shandong China; 2 Eye Hospital of Shandong, Eye Institute of Shandong First Medical University, First Medical University (Shandong Eye Hospital), 372 Jingsi Road, Jinan, 250021 China; 3 State Key Laboratory Cultivation Base, Shandong Key Laboratory of Eye Diseases, Jinan, China; 4 School of Ophthalmology, Shandong First Medical University, Jinan, China

**Keywords:** Orthokeratology lenses, Higher-order aberrations, Peripheral defocus, Myopia, Corneal topography, Eye diseases, Public health

## Abstract

This prospective study aimed to investigate changes in corneal higher-order aberrations (HOAs) in myopic children using orthokeratology (ortho-k) lenses and their relationship with myopia progression. A total of 112 children aged 8–13 years were divided into group A (axial elongation ≤ 0.1 mm/y with ortho-k) and group B (axial elongation > 0.1 mm/y with ortho-k). At baseline, 1, 6, and 12 months following the initiation of lens wear, HOAs and corneal peripheral defocus were evaluated. Ninety-three patients completed the 1-year follow-up. The mean axial elongation was − 0.07 ± 0.15 mm/y in group A, versus 0.32 ± 0.17 mm/y in group B. No statistical differences were observed in HOAs and corneal peripheral defocus at 1, 6, and 12 months (F = 0.653, 0.878; *P* > 0.05). Multivariate linear regression showed axial elongation was negatively correlated with ∆HOAs, peripheral defocus, and ∆horizontal coma (standardized beta=-0.331, -0.318, -0.209; *P* = 0.006, 0.001, 0.010, respectively) and positively correlated with the treatment zone diameter (standardized beta = 0.261, *P* = 0.003). Multivariate logistic regression identified ∆HOAs, peripheral defocus, ∆horizontal coma, and treatment zone as key factors distinguishing group A from group B (OR = 0.009, 0.455, 0.123, 12.172; *P* = 0.036, 0.003, 0.032, 0.019, respectively). The ROC curve for ∆HOAs had an area of 0.803 with a cut-off value of 0.834 μm. The ∆HOAs were more effective independent predictors of axial elongation than corneal peripheral defocus in children using ortho-k lenses. The ∆HOAs greater than 0.834 μm may lead to axial elongation ≤ 0.1 mm/y. These findings can be beneficial to fitting and optimizing ortho-k lenses.

**Supplementary Information:**

The online version contains supplementary material available at 10.1038/s41598-025-17115-w.

## Introduction

Myopia, one of the most common causes of vision impairment associated with excessive growth of axial length (AL), poses a health risk to the public, especially to children^1,2^. It is estimated that 10% of the world population will develop high myopia by 2050^3^. The risk of complications increases with the severity of myopia and AL elongation^4,5^. Therefore, it is of great significance to retard the myopia progression in children. Orthokeratology (ortho-k) is a well-documented and efficacious intervention for myopic control in pediatric patients^6,7^. However, given its higher cost and time-intensive nature compared to spectacles^8^, it is crucial to evaluate and enhance its efficacy and to explore optimal indicators for clinical use.

Ortho-k lenses correct myopia by reshaping the central cornea to be flatter and the mid-peripheral cornea to be steeper^9,10^. In myopic children wearing ortho-k lenses, the rate of 1-year axial growth delay was reported to be from 24.4–41.0%^11,12^. Ortho-k lenses with a small optical zone were disclosed to reduce the AL growth by 51.8–57%, compared to the conventional 6-mm optical zone lenses^13,14^. Despite these benefits, significant individual differences in effectiveness exist, and the underlying mechanism remains unknown. The possible factors include age, sex, spherical equivalent (SE), baseline corneal morphology, and corneal diameter^15,16^. Although they have predictive values, they are not modifiable to improve the myopia control with ortho-k.

Recent research has indicated that alterations in corneal refractive power distribution and higher-order aberrations (HOAs) following the use of ortho-k lenses are associated with AL elongation^17–19^. The ortho-k lens could help reestablish the corneal refractive power distribution, and more corneal peripheral myopic defocus may lead to slower AL elongation^20^. Hu et al.^21^ reported that the areal summed corneal power shift obtained early after ortho-k lens wear was negatively correlated with long-term AL elongation. Nevertheless, some axial growth phenomena in myopic children cannot be explained by the theory of corneal peripheral defocus. Huang et al.^22^ found that the high addition of the MSCL did not result in better myopia control efficacy. Li et al.^14^ reported that the smaller TZ (treatment zone) produced higher Zernike defocus coefficient and higher HOA after reshaping of the cornea, accompanied by better myopia control effect. However, it is still unknown which optical signal plays a dominant role. Therefore, the mechanisms underlying the prevention of myopia require further investigation. The inverse geometry corneal morphology related to ortho-k lens wear usually produces more HOAs^23,24^. The increased coma might be the most relevant factor influencing AL elongation^25^, though more clinical validation is needed. We carried out this study to investigate the connection between axial elongation and optical characteristics, such as HOAs and corneal peripheral defocus, in myopic children using ortho-k lenses, aiming to identify crucial optical factors affecting axial development. This is significant for optimizing myopia control through ortho-k, including lens fitting and design optimization, to better safeguard visual health. This study not only enriches the theoretical basis for myopia prevention and control, but also provides more possibilities for precise treatment of different populations.

## Materials and methods

### Subjects

This prospective study included patients aged from 8 to 13 years old who received ortho-k treatment for myopia at the Eye Hospital of Shandong First Medical University between January and June 2022. The observation period lasted one year (12 ± 0.5 months). Data of the right eye were used for statistical analysis. The patient inclusion and exclusion criteria are listed in Table 1. Parental written consent was obtained for every participant. The Ethics Committee of the Eye Hospital of Shandong First Medical University granted approval for this research (SDSYKYY20211219). All the procedures adhered to the tenets of the Declaration of Helsinki.

**Table 1:** Subject criteria for the study.

Inclusion criteria	Exclusion criteria
(1) Aged 8 to 13 years at baseline, with no history of ortho-k; (2) Spherical diopter from − 5.00 to -0.75 D and astigmatism > -1.50 D in both eyes (cycloplegic objective refraction); (3) Best-corrected visual acuity of 0.00 logMAR or better in both eyes; (4) Absence of active inflammation, dry eye, strabismus, and other ocular diseases; (5) No presence of diabetes, systemic immune diseases, and mental disorders; (6) Final lens parameters confirmed by both fluorescein staining and an ideal “bull’s eye” corneal pattern^26^	(1) Changes in prescription while using ortho-k lenses; (2) Discontinuation of ortho-k lens wear during the study period; (3) Not adhering to ortho-k lens wear protocols; (4) The ortho-k lens decentration more than 1.0 mm; (5) Using atropine eye drops, red light therapy devices, and other myopia intervention measures during the treatment period; (6) Known allergies to lens materials or solutions.

D, diopter; logMAR, logarithm of the minimum angle of resolution.

### General examination

Regular ocular check-ups were conducted at each follow-up, including assessments of uncorrected visual acuity (UCVA), intraocular pressure, slit-lamp microscopy, and corneal topography (Pentacam, Wavelight, Germany) for corneal parameters, the lens decentration and pupil diameter (PD)^27,28^. Axial length and curvature were measured five times consecutively using the IOL Master700 (Carl Zeiss Meditec, Germany), and the mean value was calculated. A skilled optometrist performed both objective and subjective eye exams using an auto refractometer (RM-800, Topcon, Japan) or a comprehensive refractometer (AOS-3000, Nidek, Japan), with diopters expressed as spherical equivalent (SE). All measurements were conducted between 8:00 AM and noon, approximately two hours after lens removal, to minimize diurnal variations in ocular parameters^29,30^.

#### Measurement of corneal wavefront aberrations

The wavefront aberrations of the anterior corneal surface were acquired using the Pentacam corneal topography system, with Zernike polynomial analysis^31^ performed at baseline, 1, 6, and 12 months after initiation of ortho-k lens wear. After 10 min of dark adaptation, corneal topography was conducted^32^, and three measurements with good repeatability were selected. For statistical analysis, the root mean square (RMS) values of anterior corneal surface aberrations within the central 6-mm zone, including spherical aberration Z_4_^0^, vertical coma Z_3_ ^− 1^, horizontal coma Z_3_ ^+ 1^, and HOAs, were recorded in microns (µm).

#### Measurement of corneal peripheral defocus

The differential topographic maps of the initial and subsequent follow-up tangential corneal data were extracted from the Pentacam system to assess relative corneal peripheral defocus, defined as the difference between peripheral and apex corneal refractions^22^. Measurements were taken at a 4.0-mm diameter on the nasal, temporal, superior, and inferior cornea, with the mean value calculated for statistical analysis. The treatment zone (TZ) was identified as the central flatten area encircled by points with no changes on subtractive tangential maps using a software and a best-fit ellipse technique according to the report by Guo et al.^33^.

### Ortho-k lens fitting assessment

**Fig. 1:**
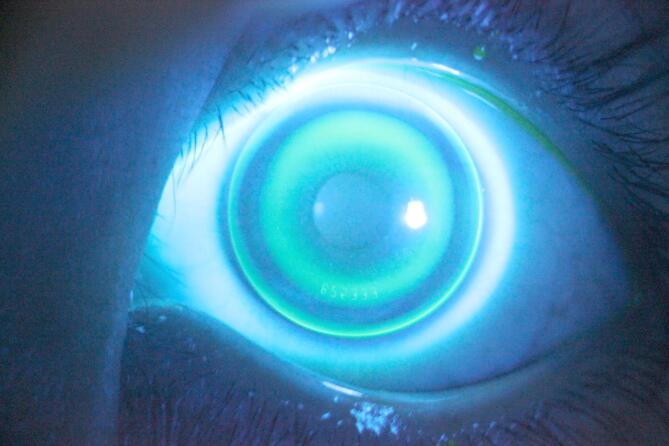
Orthokeratology lens fitting assessment using the fluorescein examination. The lens is centered, with appropriate mobility ranging from 1.0 to 1.5 mm. Each curve segment is distinct, and the base curve region measures approximately 3–5 mm in diameter. The reserve curve region forms a completely closed loop. The alignment curve fits well to the cornea, with a peripheral curve of 0.4 to 0.6 mm.

All the participants wore 3-zone ortho-k lenses (CRT, Paragon Vision Sciences, USA) with a back optic zone of 6.0 mm in diameter. A trial lens was chosen considering the corneal flat-K, corneal diameter, elevation difference at the 8-mm chord on topography, and spherical diopter. After 15 min of wear, the lens was adjusted for optimal fit based on the static and dynamic adaptation of the lens, including its position, movement, and matching of each curve, using the fluorescein staining as illustrated in Fig. 1. The overall lens diameter was designed to encompass 90–95% of the cornea. If necessary, additional modifications were performed following the true total diameter, base curvature, return zone depth, and landing zone angle. After the trial lens was removed, a corneal differential map would show that the TZ was centered, and the reverse zone was in a nearly closed loop. Moreover, the ortho-k lens decentration was assessed by measuring the distance between the center of the TZ and the center of the pupil^34,35^. Lenses were prescribed with an over-refraction goal of + 0.75 D. Patients were advised to use the lenses for 8–10 h each night, take them out in the morning, and maintain proper lens hygiene.

### Follow-up observation

Participants attended a training session 3 to 4 weeks after receiving their lenses, followed by regular follow-ups. Non-preserved saline and multifunctional solutions were used for lens disinfection. Written care instructions were given after each visit, and patients were reminded to avoid unapproved eye drops.

Follow-ups occurred at 1 day, 1 week, 1 month, and 3 months after treatment initiation, and thereafter every 3 months, with measurements of the AL taken semiannually. Additionally, the lens condition, including fit, scratches, damages, and deposits, was assessed at each visit.

### Grouping indicators

The annual AL elongation was calculated as the AL length at 1 year minus the baseline AL. The patients were divided into group A (axial elongation ≤ 0.1 mm/y with ortho-k) and group B (axial elongation > 0.1 mm/y with ortho-k)^14,36^.

### Statistical analysis

The data analyses were performed using SPSS, version 22.0 (IBM Corp., Armonk, NY, USA), available at https://www.ibm.com/analytics/spss-statistics-software. The Kolmogorov-Smirnov test was used to confirm the normal distribution of continuous data. Parametric continuous variables were presented as means ± standard deviation (SD), non-parametric continuous data as median and range, and categorical variables as counts (percentages). Differences before and after the intervention were assessed using the Paired T-test for continuous variables and the McNemar’s test for categorical variables, while differences between two groups were analyzed using the independent T-test for continuous variables and the Chi-squared test for categorical variables. Variance inflation factors (VIFs) and tolerance values were calculated to assess collinearity. A VIF < 5 and tolerance > 0.1 indicated no significant collinearity. Linear regression analysis was used to identify the explanatory variables that significantly contributed to AL elongation. Covariates adjusted in the adjusted model 1were age and sex. In model 2, the PD, AL, and SE were further adjusted based on model 1.

To further confirm the predictive power of the variables, multivariate logistic regression models were used in two groups to obtain the adjusted odd ratio (OR) and 95% confidence intervals (CI). The covariate parameters were consistent with those in linear regression. It was also necessary to determine ROC prediction cut-off points between the two groups. *P* < 0.05 was considered to be statistically significant. The sample size was determined from G*Power, version 3.1.9.7 (Heinrich-Heine-Universität Düsseldorf, Düsseldorf, Germany), available at https://gpower.software.informer.com/.

## Results

### Baseline data

**Fig. 2:**
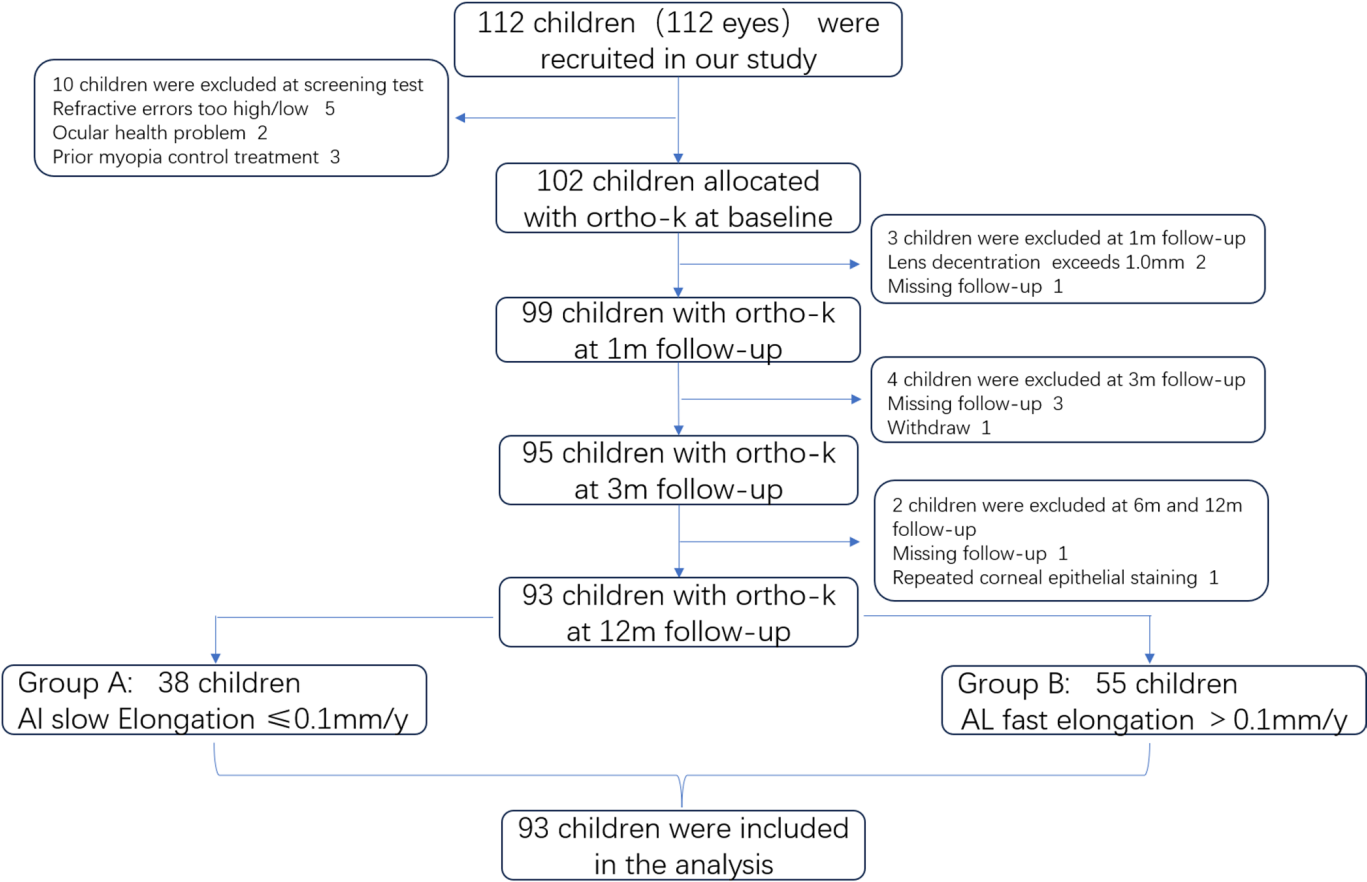
The study flow chart. The flow chart of enrolment, allocation, intervention, and assessment.

Of the 112 initially recruited patients, 93 patients (93 right eyes) completed the study. There were 53 females and 40 males, aged 9.95 ± 1.75 years. The mean SE was − 3.15 ± 1.60 D. The AL change was ≤ 0.1 mm/y in 38 eyes (group A) and > 0.1 mm/y in 55 eyes (group B) (Fig. 2). The patient demographics and biometric data at baseline are shown in Table 2. A statistically significant variation was observed in age, AL elongation, AL, and SE between the two groups. Among these variables, patients in group A were older, had a longer AL and a lower SE. No significant difference was found in sex, PD, and HOAs between groups A and B (*P* > 0.05).

**Table 2:** Demographics and biometric data of the patients at baseline.

Parameters	Mean ± SD/*n* (%)			*P* value
	All patients (*n* = 93)	Group A (38)	Group B (55)	
Age (years)	9.95 ± 1.75	10.66 ± 1.74	9.46 ± 1.60	0.004
Sex, male (n/%)	40 (43.01)	17 (44.74)	23 (41.82)	0.947
PD (mm)	3.43 ± 0.63	3.60 ± 0.73	3.31 ± 0.52	0.091
AL (mm)	24.75 ± 0.87	25.16 ± 0.89	24.46 ± 0.72	0.000
SE (D)	− 3.15 ± 1.60	− 4.32 ± 1.62	− 2.35 ± 0.99	0.000
Total aberrations (µm)	1.612 ± 0.457	1.736 ± 0.484	1.526 ± 0.422	0.094
HOAs (µm)	0.400 ± 0.185	0.394 ± 0.101	0.403 ± 0.190	0.965
Horizontal coma (µm)	0.024 ± 0.251	− 0.046 ± 0.187	0.073 ± 0.278	0.076
Vertical coma (µm)	− 0.055 ± 0.242	− 0.084 ± 0.315	0.034 ± 0.177	0.624
Spherical aberration (µm)	0.196 ± 0.082	0.218 ± 0.068	0.180 ± 0.087	0.087

*AL* axial length, *PD* pupil diameter, *SE* spherical equivalent, *HOAs* higher-order aberrations.

### Alterations in HOAs and peripheral corneal defocus with ortho-k

**Fig. 3:**
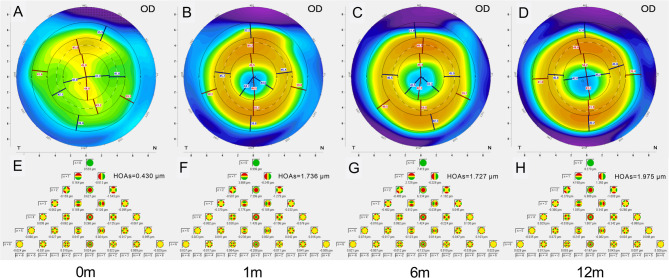
Corneal tangential topography and the Zernike aberrations terms pre- and post- ortho-k lens treatment in one patient. (**A**–**D**) the corneal tangential morphology at baseline and 1, 6, and 12 months following the initiation of treatment. (**E**–H) The aberrations of the corneal anterior surface at 0, 1, 6, and 12 months.

The morphology of the cornea continuously altered throughout the ortho-k lens shaping procedure. The HOAs of the anterior corneal surface continued to rise at 1, 6, and 12 months of ortho-k lens wear, accompanied by corneal deformation (Fig. 3). Notable statistical variations were observed in overall HOAs, horizontal coma, and spherical aberration pre- and post-ortho-k lens usage. The corneal peripheral defocus showed no significant difference at 1, 6, and 12 months, the same as the HOAs. These changes in HOAs and corneal peripheral defocus in all patients during the follow-up period are presented in Table 3.

**Table 3:** Alterations in corneal peripheral defocus, hoas, and TZ diameter in the corneal anterior surface pre- and post- ortho-k lens treatment (mean ± SD).

Time	Corneal peripheral defocus (D)	HOAs (µm)	Horizontal coma (µm)	Vertical coma (µm)	Spherical aberration (µm)	TZ diameter
0d	NA	0.400 ± 0.185	0.024 ± 0.251	− 0.055 ± 0.242	0.196 ± 0.082	NA
1 m	7.53 ± 1.98	1.173 ± 0.345*	0.390 ± 0.486*	− 0.048 ± 0.522	0.742 ± 0.200*	2.656 ± 0.465
6 m	7.69 ± 2.10	1.227 ± 0.393*	0.414 ± 0.549*	0.011 ± 0.600	0.730 ± 0.176*	2.851 ± 0.506
12 m	7.88 ± 2.17	1.238 ± 0.333*	0.451 ± 0.486*	0.004 ± 0.606	0.720 ± 0.177*	2.907 ± 0.477
F	0.653	150.874	17.515	0.402	245.457	6.933
*P*-value	0.521	0.000	0.000	0.751	0.000	0.001

*HOAs* higher-order aberrations, *TZ* treatment zone, *NA* no applicable.

*Indicates statistical difference from baseline.

### Linear regression analyses of axial elongation and ocular parameters with ortho-k

The linear multivariate regression analysis in fully adjusted model 2 showed significant correlations of ∆HOAs (standardized beta= -0.331, *P* = 0.006), corneal peripheral focus (standardized beta= -0.318, *P* = 0.001), ∆horizontal coma (standardized beta= -0.209, *P* = 0.010), and TZ size (standardized beta = 0.261, *P* = 0.003), with ∆HOAs having the greatest impact (Table 4). The univariate linear analysis results are provided in the supplementary data 1.

### Logistic regression results and ROC curve of axial elongation and ocular parameters between two groups

**Fig. 4:**
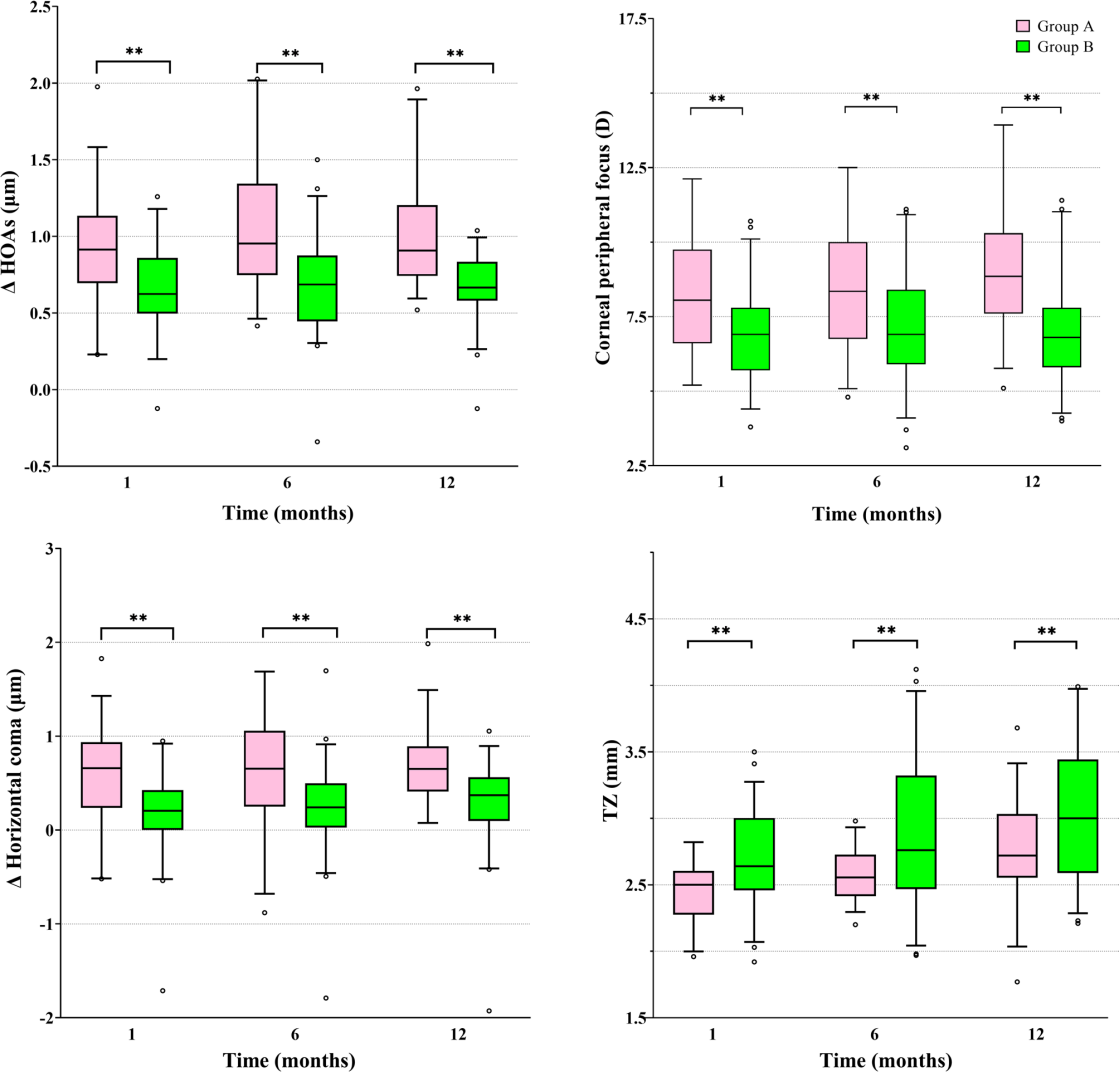
Comparative analysis of the ∆HOAs, ∆horizontal coma, corneal peripheral defocus, and TZ box plot between two groups over time. There were statistical differences between two groups at 1, 6, and 12 months, but no significant differences between different time points within each group. ** *P* < 0.01. Pink represents group A; green represents group B. HOAs, Higher-order aberrations; TZ, treatment zone.

The multivariable logistic regression model 2 indicated ∆HOAs (OR = 0.009, *P* = 0.036), corneal peripheral focus (OR = 0.455, *P* = 0.003), ∆ horizontal coma (OR = 0.123, *P* = 0.032), and TZ size (OR = 12.172, *P* = 0.019) were the key parameters to distinguish group A from group B (Table 5; Fig. 4). The univariate logistic regression results are shown in the supplementary data 2.

**Fig. 5:**
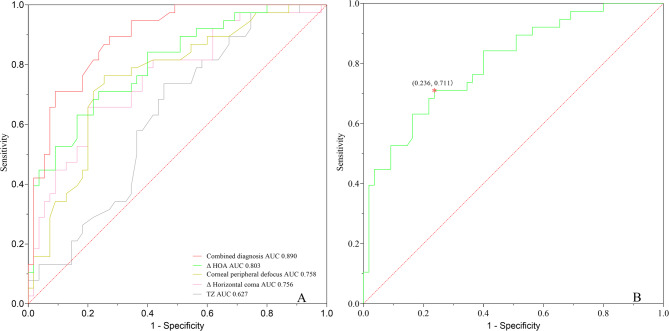
Receiver Operating Characteristic (ROC) curves for the prediction of rapid (> 0.1 mm/year) versus slow (≤ 0.1 mm/year) axial length (AL) elongation. A, ROC curves of ∆HOAs, corneal peripheral defocus, ∆ horizontal coma, TZ, and combined diagnosis. B, ROC curve of ∆HOAs, with a threshold value of 0.834 μm. ROC, receiver operating characteristic; HOAs, high-order aberrations; TZ, treatment zone.

The ROC prediction cut-off points between the two groups were explored by employing individual factors as predictors to calculate the area under the ROC curve (AUC), yielding values of 0.803 for ∆HOAs, 0.758 for corneal peripheral defocus, 0.756 for ∆ horizontal coma, and 0.627 for TZ. The most reliable independent indicator of axial elongation was ∆HOAs, with a cut-off value of 0.834 μm. Moreover, the multivariable stepwise logistic regression analysis was performed to further distinguish group A from group B using the four factors. The predictive performance was significantly improved (AUC = 0.890) (Fig. 5).

**Table 4:** Multivariate linear regression analyses of AL elongation and ocular parameters with ortho-k.

Parameter	Unadjusted model*			Adjusted model 1*			Adjusted model 2*		
	Standardized beta	Beta (95% CI)	*P*-value	Standardized beta	Beta (95% CI)	*P*-value	Standardized beta	Beta (95% CI)	*P*-value
∆ Total aberration	0.085	0.012 (− 0.028 to 0.053)	0.552	0.211	0.019 (− 0.014 to 0.062)	0.167	0.192	0.027 (− 0.009 to 0.064)	0.142
∆ HOAs	− 0.366	− 0.260 (− 0.430 to − 0.090)	0.003	− 0.283	− 0.202 (− 0.360 to − 0.043)	0.013	− 0.331	− 0.236 (− 0.403 to − 0.068)	0.006
∆ horizontal coma	− 0.284	− 0.132 (− 0.210 to − 0.055)	0.001	− 0.262	− 0.122 (− 0.194 to − 0.051)	0.001	− 0.209	− 0.097 (− 0.171 to − 0.024)	0.010
corneal peripheral defocus	− 0.287	− 0.033 (− 0.056 to − 0.010)	0.006	− 0.364	− 0.042 (− 0.064 to − 0.020)	0.000	− 0.318	− 0.037 (− 0.058 to − 0.015)	0.001
TZ diameter	0.275	0.145 (0.048 to 0.243)	0.004	0.294	0.155 (0.066 to 0.245)	0.001	0.261	0.138 (0.050 to 0.226)	0.003

*CI* confidence interval, *PD* pupil diameter, *AL* axial length, *HOAs* higher-order aberrations, *TZ* treatment zone.

*In adjusted model 1, age and sex were adjusted; in adjusted model 2, PD, AL, and SE were adjusted based on model 1.

**Table 5:** Multivariate logistic regression analyses of AL elongation and ocular parameters with ortho-k.

Parameter	Unadjusted model^*^			Adjusted model 1^*^			Adjusted model 2^*^		
	B	OR (95% CI)	*P*-value	B	OR (95% CI)	*P*-value	B	OR (95% CI)	*P*-value
∆ Total aberration	0.492	1.635 (0.767 to 3.487)	0.203	0.747	2.112 (0.909 to 4.905)	0.082	0.790	2.204 (0.873 to5.567)	0.094
∆ HOAs	− 4.228	0.015 (0.001 to 0.420)	0.014	− 4.435	0.012 (0.000 to 0.530)	0.022	− 4.690	0.009 (0.000 to 0.730)	0.036
∆ horizontal coma	− 2.469	0.085 (0.016 to 0.444)	0.004	− 2.634	0.072 (0.012 to0.428)	0.004	− 2.093	0.123 (0.018 to0.838)	0.032
corneal peripheral defocus	− 0.533	0.587 (0.390 to 0.884)	0.011	− 0.789	0.454 (0.2814 to 0.734)	0.001	− 0.788	0.455 (0.270 to 0.766)	0.003
TZ diameter	2.075	7.966 (1.552 to 40.872)	0.013	2.807	16.560 (2.689 to 102.00)	0.002	2.499	12.172 (1.499 to 98.811)	0.019

*CI* confidence interval, *PD* pupil diameter, *AL* axial length, *HOAs* higher-order aberrations, *TZ* treatment zone, *OR* odd ratio.

* In adjusted model 1, age and sex were adjusted; in adjusted model 2, PD, AL, and SE were adjusted based on model 1.

## Discussion

During the process of ortho-k lens wear, corneal reshaping causes peripheral myopia defocus of the cornea^37,38^ and increase corneal HOAs^39^which may be potential mechanisms to control myopia, but no consensus opinions have been formed. In this study, ∆HOAs, corneal peripheral myopic defocus, ∆horizontal coma, and TZ diameter were disclosed as the most significant factors correlated with AL elongation, among which ∆HOAs presented the greatest impact. When the ∆HOAs exceeded 0.834 μm, the probability of axial elongation no more than 0.1 mm/y was 67.5%. Previous studies revealed significant changes in overall HOAs, spherical aberrations, and coma following ortho-k lens usage^25,39^. However, Lau et al.^40^ reported a negative correlation between spherical aberration and AL elongation, which differed from our discovery that the ortho-k treatment contributed to higher spherical aberration, but had no correlation with the AL elongation. The reasons may be associated with the differences in measurement methods and diameter ranges used for HOA assessment. The acquired data in the current series were fitted with a sixth-order Zernike polynomial using a 6-mm PD, displaying a negative correlation between HOAs, horizontal coma, and axial growth, which was similar to the findings of Li et al.^41^ The findings of our study suggest that higher-order aberrations (HOAs) may serve as useful indicators for the early assessment of ortho-k lens efficacy following intervention. These results can help clinicians refine ortho-k lens design and improve treatment outcomes. We recommend that practitioners routinely measure HOAs during follow-up visits to monitor treatment progress. ΔHOAs can be readily extracted from corneal topography maps. In the future, aggregating more HOA data may support the development of early evaluation protocols and personalized lens designs.

In this research, a threshold of 0.1 mm per year was employed for classification, primarily because 0.1 mm represents the natural range of axial growth in children aged 8 to 13, making it effective for differentiation between slow and fast axial growth groups. It is very interesting to show an average annual growth of -0.07 mm in the slow growth group, which challenges the previous understanding that the AL cannot be shortened^42^. One possible explanation for this phenomenon is that ortho-k decreases the central corneal thickness while increasing the choroidal thickness^43,44^. Notably, achieving an annual axial growth ≤ 0.1 mm is likely to result in highly favorable clinical outcomes. Research has been conducted to evaluate the characteristics of relative peripheral defocus^21,45^, such as the spatial distribution of changes in relative corneal refractive power and their connections to axial lengthening^46,47^. It is hypothesized that alterations in relative peripheral myopic defocus closer to the corneal center might enhance myopia control, explaining how ortho-k slows the progression of myopia. The subjects in our study demonstrated satisfactory corneal reshaping, with a decentration of less than 1.0 mm. Meanwhile, the corneal peripheral defocus was within a diameter of 4.0 mm and not less than 3.8 D. However, 59.1% (55/93) of children were in the fast-growing group, suggesting that the theory of myopic defocus alone is insufficient to explain this clinical observation. There should be additional control mechanisms influencing AL elongation. In the current series, approximately 9.7% (9/93) of ortho-k lenses could be absolutely centered, whereas the other majority experienced varying degrees of eccentricity, particularly temporal decentration. These findings indicate that temporal eccentric shaping and asymmetric corneal shapes would introduce more HOAs and horizontal coma, thereby increasing the efficiency of myopia control. At present, the optimization of ortho-k lens design is actively being integrated into clinical practice. This process involves modifications such as incorporating an aspherical design for the base curve or reducing the base curve zone of the lens, which may potentially enhance HOAs upon ocular entry^48,49^. However, further clinical research is required to quantify the extent of changes in HOAs resulting from these modifications.

In this study, 38 patients (40.9%, 38/93) whose annual AL elongation was ≤ 0.1 mm achieved excellent control effects during the process of ortho-k lens treatment, while 55 patients (59.1%, 55/93) still experienced AL growth above 0.1 mm per year, including 25 patients (26.9%, 25/93) with AL growth above 0.3 mm per year. The differentiation of myopia control effects has always existed, which is highly related to the complexity of myopia pathogenesis and inconsistent treatment response. Further studies are needed to clarify the pathogenesis of myopia and find more targeted control directions. Our study further confirms the critical significance of ∆HOAs in controlling myopia with ortho-k, which is beneficial for better understanding the differentiation of clinical efficacy.

Multiple regression models were established in the current study and demonstrated that the smaller the TZ diameter, the better the control effect, which is consistent with the findings of previous studies^50,51^. Guo et al.^52^ and Li et al.^41^ reported that the ortho-k lenses with a 5-mm optic zone can reduce AL elongation by 53.6% and 76.5%, respectively, compared to those with a larger optic zone. In our series, customized lenses with a 6-mm optic zone may form different TZ sizes after ortho-k lens wear in different people, which may be attributed to the individual fitting of the lens and specific corneas. This study suggests that better myopia control can be achieved by reducing the TZ. Furthermore, no notable disparity was found in PD between the two groups and no clear link between PD and myopia management. The cause could be that the immediate scotopic PD recorded was inadequate to represent the pupil size of children in their everyday activities. Therefore, the PD did not influence AL elongation, which contradicts the findings of Pauné et al.^51^.

In this study, the ∆HOAs showed significant correlations with AL elongation and generated the largest area under the ROC as compared to corneal peripheral myopic defocus, ∆ horizontal coma, and TZ diameter. Therefore, the ∆HOAs can be used as the best independent predictors for AL elongation. This finding may enhance the method of managing myopia with ortho-k lenses, aiding in forecasting myopia control outcomes and refining the ortho-k lens design. Furthermore, there was no significant difference in ∆HOAs at 1, 6, and 12 months with ortho-k. Therefore, based on the change in ∆HOAs at 1 month, an early prediction of axial growth might be made.

Our study has limitations. First, the corneal peripheral defocus was examined in myopes treated with ortho-k, which cannot truly reflect the relationship between retinal peripheral defocus and myopia growth. Second, the sample size was comparatively small, and the observation time was short. Third, considering the etiology complexity of myopia^53^, such as outdoor activities, near work, diet and lifestyle habits, the efficacy of ortho-k lens in myopia control cannot be reliably predicted by specific models. Nevertheless, to improve the effectiveness of future strategies for myopia prevention and control, it is essential to actively promote outdoor activities, reduce near work, and encourage healthy dietary habits^54,55^. Prospective investigations with a larger sample size, a longer observation period, and more lens designs are required to further explore the mechanisms and feasible evaluation methods.

## Conclusion

In conclusion, ortho-k lens treatment with higher ∆HOAs may lead to better efficacy of myopia control. The ∆HOAs diagnosis ROC may be helpful for evaluating myopia control effects and adjusting the ortho-k lens parameters in time, so as to achieve greater AL retardation benefits. If the ∆HOAs exceed 0.834 μm, there is a 67.5% probability that the axial elongation is no more than 0.1 mm per year.

## Supplementary Information

Below is the link to the electronic supplementary material.

Supplementary Material 1

Supplementary Material 2

## Data Availability

The data that support the findings of the current study are available from the corresponding author upon reasonable request.
