# Supplementary material for: Corneal higher-order aberrations as key predictive indicators of axial elongation in myopic children with orthokeratology: a single-center prospective cohort study
Source: Sci Rep. 2025 Aug 23;15:31065. doi: 10.1038/s41598-025-17115-w (PMC12375064; doi:10.1038/s41598-025-17115-w)
Supplement: Supplementary file 1 — Supplementary Material 1 [file 41598_2025_17115_MOESM1_ESM.docx]

| Table 2. Univariate logistic regression analyses of AL elongation and ocular parameters between two groups after ortho-k lens treatment | | | |
| --- | --- | --- | --- |
| Parameters | Univariate regression | | |
|  | B | OR (95% CI) | *P*-value |
| Age (years) | -0.415 | 0.660 (0.510 to 0.854) | 0.002 |
| Sex | 0.119 | 1.126 (0.489 to 2.954) | 0.780 |
| PD (mm) | -0.759 | 0.468 (0.234 to 0.936) | 0.032 |
| AL (mm) | -1.115 | 0.328 (0.178 to 0.606) | 0.000 |
| SE (D) | 1.028 | 2.796 (1.852 to 4.223) | 0.000 |
| Δ total aberration | -0.693 | 0.500 (0.355 to 0.706) | 0.000 |
| Δ HOAs | -4.418 | 0.012 (0.001 to 0.118) | 0.000 |
| Δ vertical coma | -0.437 | 0.646 (0.395 to1.058) | 0.082 |
| Δ horizontal coma | -1.969 | 0.140 (0.043 to 0.457) | 0.001 |
| Δ spherical aberration | 0.003 | 1.003 (0.101 to9.916) | 0.998 |
| corneal peripheral focus | -0.495 | 0.609 (0.476 to 0.780) | 0.000 |
| TZ diameter | 1.232 | 3.427 (1.274 to 9.219) | 0.015 |
| CI, confidence interval; OR, odds ratio; PD, pupil diameter; AL, axial length; HOAs, high-order aberrations; TZ, treatment zone. | | | |
