# Supplementary material for: Corneal higher-order aberrations as key predictive indicators of axial elongation in myopic children with orthokeratology: a single-center prospective cohort study
Source: Sci Rep. 2025 Aug 23;15:31065. doi: 10.1038/s41598-025-17115-w (PMC12375064; doi:10.1038/s41598-025-17115-w)
Supplement: Supplementary file 2 — Supplementary Material 2 [file 41598_2025_17115_MOESM2_ESM.docx]

| Table 1. Univariate linear regression analyses of AL elongation and ocular parameters after ortho-k lens treatment | | | | | | |
| --- | --- | --- | --- | --- | --- | --- |
| Parameters | Univariate regression | | | | | |
|  | | R | Standardized beta | F | Beta (95% CI) | *P*-value |
| Age (years) | | 0.441 | -0.441 | 21.968 | -0.063 (-0.090 to -0.037) | 0.000 |
| Sex | | 0.038 | 0.038 | 0.131 | 0.019 (-0.086 to 0.125) | 0.718 |
| PD (mm) | | 0.262 | -0.262 | 6.733 | -0.105 (-0.186 to -0.025) | 0.011 |
| AL (mm) | | 0.453 | -0.453 | 23.433 | -0.132 (-0.186 to -0.078) | 0.000 |
| SE (D) | | 0.541 | 0.541 | 37.695 | 0.085 (0.058 to 0.113) | 0.000 |
| Δ Total aberration | | 0.552 | -0.552 | 39.833 | -0.076 (-0.101 to -0.051) | 0.000 |
| Δ HOAs | | 0.554 | -0.554 | 40.329 | -0.395 (-0.518 to -0.271) | 0.000 |
| Δ vertical coma | | 0.07 | -0.07 | 0.445 | -0.024 (-0.094 to -0.047) | 0.506 |
| Δ horizontal coma | | 0.39 | -0.39 | 16.346 | -0.182 (-0.271 to -0.092) | 0.000 |
| Δ spherical aberration | | 0.013 | -0.013 | 0.016 | -0.019 (-0.308 to 0.271) | 0.899 |
| corneal peripheral defocus | | 0.493 | -0.493 | 29.215 | -0.057 (-0.078 to -0.036) | 0.000 |
| TZ diameter | | 0.302 | 0.302 | 9.157 | 0.160 (0.055 to 0.265) | 0.003 |
| CI, confidence interval; PD, pupil diameter; AL, axial length; HOAs, high-order aberrations; TZ, treatment zone. | | | | | | |
